# The nomogram prediction model for primary aldosteronism: the combination of serum and urine electrolytes and serum uric acid

**DOI:** 10.3389/fendo.2026.1875217

**Published:** 2026-09-21

**Authors:** Xiaoyu Zha, Yanv Ren, Hao Wang, Zuoliang Dong

**Affiliations:** 1 Department of Clinical Laboratory, Tianjin Medical University General Hospital, Tianjin, China; 2 Department of Endocrinology and Metabolism, Tianjin Medical University General Hospital, Tianjin, China

**Keywords:** essential hypertension, nomogram, prediction model, primary aldosteronism, secondary hypertension

## Abstract

**Purpose:**

To construct and preliminarily validate a novel nomogram for primary aldosteronism (PA) risk estimation, with the goal of providing a potential reference tool for PA screening in clinical practice.

**Methods:**

Hypertensive inpatients who underwent PA screening at the Department of Endocrinology and Metabolism, Cardiology, and Urology of Tianjin Medical University General Hospital from October 2021 to July 2024 were included. Basic information and laboratory test results of the patients were collected, and were randomly divided into training set and validation set at a 7:3 ratio. Differential analysis, Lasso regression, univariate logistic regression, and multivariate logistic regression were used to screen the variables associated with PA. The model results were presented as a nomogram. Predictive ability was assessed by plotting receiver operating characteristic (ROC) curves, and the diagnostic performance of the model was compared using the DeLong test. The Hosmer-Lemeshow test was used to evaluate the goodness of fit of the regression model. The clinical utility of the model was evaluated by clinical decision curve analysis (DCA). *Kappa* test was used to evaluate the agreement between the model and ARR.

**Results:**

Six indicators including serum sodium-to-potassium ratio, anion gap, uric acid, urine sodium, 24-hour urine potassium and urine pH were used to construct a nomogram prediction model for PA. The training and validation sets achieved area under the curve (AUC) values of 0.860 (95% CI: 0.825–0.894) and 0.850 (95% CI: 0.800–0.899), respectively. The AUCs of the validation set for PFK score and ARR were 0.718 (95% CI: 0.656–0.781) and 0.965 (95% CI: 0.943–0.987), respectively. The model demonstrated good discrimination ability. The Hosmer-Lemeshow test indicated a good fit (training set: *χ²* = 7.897, *P* = 0.444;validation set: *χ²* = 6.208, *P* = 0.624). The DCA showed that the model had good clinical application value. The *Kappa* test showed good agreement between the model and ARR(*Kappa* = 0.364, *P* < 0.001).

**Conclusion:**

The nomogram model based on serum sodium-to-potassium ratio, anion gap, uric acid, urine sodium, 24-hour urine potassium and urine pH can predict the risk of PA, facilitating PA screening in clinical practice.

## 1 Introduction

Primary aldosteronism (PA) is one of the common causes of secondary hypertension. It was first described by American physician Jerome W. Conn in 1955, who reported a case of hypertension caused by an aldosterone-producing adrenal adenoma (1, 2). Among the hypertensive population, the prevalence of PA ranges from approximately 10% to 20%. Specifically, the prevalence rates of PA among individuals with high-normal blood pressure, Stage 1, Stage 2, and Stage 3 hypertension were 5.5%, 4.2%, 10.2%, and 16.4%, respectively. Among hypertensive patients in primary care, the estimated prevalence of PA ranges from 3.2% to 12.7% (3). Studies have shown that, compared to patients with essential hypertension (EH), those with PA exhibit significantly increased risks of atrial fibrillation, stroke, heart failure, and coronary artery disease (4). Unlike EH, however, PA has identifiable causes, and most patients can be cured through surgery or drug therapy. Therefore, early screening and diagnosis of PA are particularly important. Indeed, the Mayo Clinic recommends that all hypertensive patients undergo at least one PA screening (5). Despite this recommendation, PA screening rates remain low both in China and in other countries. A survey conducted in China revealed that only 0.1% of primary care hospitals conduct PA screening (6). International studies report that only 7%–8% of hypertensive patients have undergone PA screening. Even among those with hypokalemia, only 2.7% have been screened for PA (7, 8). The 2025 American Endocrine Society Clinical Practice Guidelines for PA recommend screening all hypertensive patients to prevent PA from being misdiagnosed as EH and to avoid missing targeted treatment opportunities (9).The aldosterone-to-renin ratio (ARR), defined as the ratio of plasma aldosterone concentration (PAC) to direct renin concentration (DRC), is currently the internationally recognized screening indicator for PA (10, 11). Our previous research established the optimal ARR cutoff point for PA screening in Tianjin, China (12). However, the use of ARR faces several practical limitations. First, patients must discontinue medications that affect ARR—such as aldosterone receptor antagonists, potassium-sparing diuretics, and angiotensin-converting enzyme inhibitors—prior to PA testing, which is significantly inconvenient for patients. Second, since aldosterone and renin testing predominantly relies on chemiluminescence methods that require specialized equipment, most primary care hospitals lack the necessary testing capabilities (6). Collectively, these factors limit the widespread adoption of ARR. Consequently, there is an urgent clinical need for a new, easily implementable PA screening tool.

To address this need, the present study developed a nomogram-based prediction model for PA. Specifically, we analyzed relevant serum and urine indicators in patients with EH and PA. Using Lasso regression and logistic regression methods, we established a PA prediction nomogram based on serum sodium-to-potassium ratio, anion gap, uric acid, urine sodium, 24-hour urine potassium, and urine pH. This model may serve as a foundation for developing more user-friendly tools for primary care settings in the future.

## 2 Materials and methods

### 2.1 Patient population

Hypertensive inpatients who underwent PA screening at the Department of Endocrinology and Metabolism, Cardiology, and Urology of Tianjin Medical University General Hospital from October 2021 to July 2024 were retrospectively enrolled. Patients were included if they had the following parameters measured: PAC, DRC, ARR, serum sodium, serum potassium, serum chloride, serum calcium, anion gap, total cholesterol, triglycerides, blood glucose, urea, creatinine, uric acid, urine sodium, urine potassium, and urine pH. The screening criteria for PA were based on the *Expert Consensus on Diagnosis and Treatment of Primary Aldosteronism (2024 Edition)* (13). Main criteria included: 1) Patients with persistent hypertension (BP > 150/100 mmHg, 1 mmHg = 0.133 kPa); patients whose blood pressure remained uncontrolled (≥ 140/90 mmHg) despite treatment with three conventional antihypertensive agents (including diuretics); patients requiring ≥ 4 antihypertensive agents to achieve control (< 140/90 mmHg); and newly diagnosed hypertensive patients; 2) Hypertensive patients with spontaneous or diuretic-induced hypokalemia; 3) Hypertensive patients with incidentally discovered adrenal tumors; 4) Hypertensive patients with a family history of early-onset hypertension or early-onset (age < 40 years) cerebrovascular accident; 5) Hypertensive first-degree relatives of patients with PA; 6) Hypertensive patients with obstructive sleep apnea. Exclusion criteria were: other types of secondary hypertension (secondary hyperaldosteronism, renal artery stenosis, Cushing’s syndrome, pheochromocytoma,etc.), stage IV heart failure, severe organic heart disease, severe arrhythmia, severe hepatic or renal impairment, pregnancy or lactation.

### 2.2 Data collection

Patient demographics were collected, including gender, age, body mass index, blood pressure, presence of diabetes, and hypokalemia.The initial laboratory results obtained upon admission included: PAC, DRC, ARR (LIAISON^®^XL fully automated chemiluminescent immunoassay analyzer, DiaSorin Italia S.p.A.); serum sodium, potassium, chloride, calcium, anion gap, total cholesterol, triglycerides, glucose, urea, creatinine, and uric acid (008AS fully automated biochemical analyzer, Hitachi High-Tech Corporation); urine sodium concentration, 24-hour urine sodium, urine potassium concentration, and 24-hour urine potassium (7180 fully automated biochemical analyzer, Hitachi High-Tech Corporation); urine pH (UF-1000i fully automated urine analyzer, Sysmex Corporation).

### 2.3 ARR screening

ARR screening was conducted according to the following standardized protocol:

1) Sampling conditions: Blood samples were collected in the morning (between 8:00 AM and 10:00 AM) after patients had been ambulatory (sitting, standing, or walking) for at least 2 hours, followed by 5–15 minutes of seated rest before collection. All samples were drawn with the patient in a seated position. 2) Potassium correction: Serum potassium levels were corrected to the normal range (≥ 3.5 mmol/L) before ARR measurement, as hypokalemia can suppress renin and falsely elevate ARR. 3) Assay methods and ARR cutoff: PAC and DRC were measured using chemiluminescent immunoassay. The ARR was calculated as PAC/DRC. An ARR ≥ 3.70 with PAC > 15 ng/dL was considered screening-positive. 4) Medication adjustment: To minimize the influence of antihypertensive medications on ARR results, patients were instructed to discontinue medications that significantly affect the renin-angiotensin-aldosterone system when clinically feasible. Specifically, mineralocorticoid receptor antagonists (e.g., spironolactone, eplerenone), potassium-sparing diuretics, potassium-wasting diuretics, and licorice extract were withdrawn for at least 4 weeks prior to testing. Angiotensin-converting enzyme inhibitors (ACEIs), angiotensin receptor blockers (ARBs), and calcium channel blockers (CCBs) were withdrawn for at least 2 weeks prior to testing. For patients in whom complete withdrawal was not feasible, antihypertensive therapy was switched to agents with minimal effects on ARR, such as slow-release verapamil, hydralazine, prazosin, or doxazosin (11).

### 2.4 PA and EH diagnostic criteria

The diagnostic criteria for PA were as follows. Patients with a positive ARR underwent confirmatory testing using either a captopril suppression test or a saline loading test. PA was confirmed if the captopril suppression test showed an aldosterone suppression rate of ≤ 30% at 2 hours or a PAC > 11 ng/dL, or if the saline loading test showed a PAC > 10 ng/dL. Patients with concomitant spontaneous hypokalemia, plasma renin levels below the detectable limit, and aldosterone levels > 20 ng/dL were directly diagnosed with PA without additional confirmatory tests (11). EH was diagnosed by excluding the following secondary causes of hypertension: renal parenchymal hypertension (e.g., IgA nephropathy, membranoproliferative glomerulonephritis, polycystic kidney disease, chronic pyelonephritis, obstructive nephropathy, lupus nephritis), renal artery stenosis, aortic stenosis, PA, pheochromocytoma/paraganglioma, Cushing’s syndrome, Liddle syndrome, Gordon syndrome, apparent mineralocorticoid excess syndrome, and other secondary causes.

### 2.5 Statistical analysis

Statistical analyses were performed using the R software package (R version 4.3.3). Missing values were imputed using the PMM method. Clinical baseline data were analyzed according to data type. Normally distributed continuous variables were expressed as mean ± standard deviation and were compared between groups using the independent samples t-test. Non-normally distributed continuous variables were expressed as median (interquartile range) and compared between groups using the Mann-Whitney U test. Categorical variables were expressed as frequencies and percentages, with intergroup comparisons performed using the χ² test. Variables associated with PA were identified using Lasso regression, univariate logistic regression, and multivariate logistic regression. Statistical significance was set at *P* < 0.05. The model results were presented as a nomogram. Receiver operating characteristic (ROC) curves were plotted to evaluate the predictive ability of the model. The Hosmer-Lemeshow test was used to assess the goodness of fit of the regression model, with *P* > 0.05 indicating adequate fit. DCA was used to evaluate the clinical utility of the model. The *Kappa* statistic was used to measure the agreement between the model and ARR screening results for PA, with P < 0.05 considered statistically significant.

## 3 Results

### 3.1 Basic clinical characteristics of study subjects

A total of 1601 patients were included in this study, comprising 1349 cases of EH and 252 cases of PA. These patients were randomly assigned to a training set (1120 cases) and a validation set (481 cases) at a ratio of 7:3. No statistically significant differences were observed between the training and validation sets in terms of serum sodium-to-potassium ratio, serum chloride, serum calcium, anion gap, total cholesterol, triglycerides, blood glucose, urea, creatinine, uric acid, or other indicators (*P* > 0.05), indicating that the sample populations were balanced and comparable (**Table 1**).

**Table 1:** Baseline characteristics of training and validation sets.

Variables	Training set (n = 1120)	Validation set (n = 481)	*t/Z/χ²*	*P*
Age (years)	53.00 (40.00, 64.00)	53.00 (40.00, 63.00)	-0.697	0.486
Gender [n (%)]				
Male	578 (51.61)	248 (51.56)	<0.001	0.986
Female	542 (48.39)	233 (48.44)		
BMI (kg/m^2^)	26.52 (24.06, 29.75)	26.42 (24.15, 29.41)	-0.155	0.877
SBP (mmHg)	143.00 (130.00, 155.00)	143.00 (130.00, 156.00)	-0.126	0.900
DBP (mmHg)	89.00 (80.00, 100.00)	89.00 (80.00, 99.00)	-0.402	0.688
PAC (ng/dL)	13.20 (8.40, 19.62)	12.50 (8.60, 20.20)	-0.238	0.812
DRC (μ IU/mL)	16.90 (5.80, 46.08)	17.60 (5.10, 47.90)	-0.122	0.903
ARR [(ng/dL)/(μ IU/mL)]	0.68 (0.27, 2.28)	0.64 (0.24, 2.36)	-0.233	0.815
Serum Na (mmol/L)	142.00 (141.00, 144.00)	142.00 (141.00, 143.00)	-0.986	0.324
Serum K (mmol/L)	3.80 (3.50, 4.10)	3.80 (3.50, 4.00)	-0.867	0.386
Serum Na-to-K ratio	37.37 (34.88, 40.86)	37.63 (35.00, 40.57)	-0.660	0.509
Serum Cl (mmol/L)	105.00 (104.00, 107.00)	105.00 (103.00, 107.00)	-1.118	0.263
Serum Ca (mmol/L)	2.30 (2.22, 2.36)	2.30 (2.22, 2.37)	-0.122	0.903
AG	13.90 (12.30, 15.50)	14.00 (12.50, 15.60)	-0.923	0.356
TC (mmol/L)	4.68 (4.03, 5.41)	4.80 (4.09, 5.30)	-0.763	0.445
TG (mmol/L)	1.64 (1.14, 2.30)	1.65 (1.23, 2.49)	-1.698	0.089
GLU (mmol/L)	5.00 (4.50, 5.80)	5.10 (4.60, 5.80)	-1.210	0.226
UREA (mmol/L)	4.80 (4.10, 5.70)	4.80 (4.00, 5.70)	-0.803	0.422
CREA (μmol/L)	64.00 (54.00, 75.00)	63.00 (52.00, 76.00)	-0.692	0.489
UA (μmol/L)	344.00 (282.75, 421.00)	347.00 (279.00, 428.00)	-0.561	0.575
Urine Na (mmol/L)	80.05 (53.58, 110.38)	78.90 (51.90, 109.70)	-0.497	0.619
24h Urine Na (mmol/24h)	151.55 (103.19, 215.26)	156.90 (112.70, 227.70)	-1.743	0.081
Urine K (mmol/L)	20.09 (15.03, 28.74)	19.76 (14.41, 27.82)	-0.866	0.386
24h Urine K (mmol/24h)	39.66 (29.55, 51.63)	41.27 (31.40, 54.50)	-1.864	0.062
Urine pH	6.00 (5.50, 6.50)	6.00 (5.50, 6.50)	-1.021	0.307
Diabetes [n (%)]				
Yes	251 (22.41)	113 (23.49)	0.224	0.636
No	869 (77.59)	368 (76.51)		
Hypokalemia [n (%)]				
Yes	175 (15.62)	80 (16.63)	0.255	0.614
No	945 (84.38)	401 (83.37)		
Diagnosis [n (%)]				
PA	174 (15.54)	78 (16.22)	0.117	0.732
EH	946 (84.46)	403 (83.78)		

### 3.2 Screening of predictors for the PA nomogram prediction model

Differential analysis was performed on the following variables in the training set: age, gender, BMI, blood pressure, serum sodium, serum potassium, serum sodium-to-potassium ratio, serum calcium, anion gap, total cholesterol, triglycerides, blood glucose, urea, creatinine, and uric acid. The results showed that systolic blood pressure, diastolic blood pressure, serum sodium, serum sodium-to-potassium ratio, urine potassium, 24-hour urine potassium and urine pH were significantly higher in the PA group than in the EH group (*P* < 0.05). Additionally, the proportion of patients with hypokalemia in the PA group was higher than in the EH group (*P* < 0.05). Conversely, the PA group exhibited lower levels of BMI, serum potassium, serum calcium, anion gap, total cholesterol, triglycerides, blood glucose, urea, uric acid, and urine sodium compared with the EH group (*P* < 0.05) (**Table 2**). These statistically significant indicators were included in the Lasso regression, and six indicators including serum sodium-to-potassium ratio, anion gap, uric acid, urine sodium, 24-hour urine potassium, and urine pH were screened as potential predictors of the PA prediction model (**Figure 1**).

**Table 2:** Differential analysis of the training set.

Variables	PA (n = 174)	EH (n = 946)	*t/Z/χ²*	*P*
Age (years)	54.00 (44.25, 63.00)	52.00 (39.00, 65.00)	-1.447	0.148
Gender [n (%)]				
Male	86 (49.43)	492 (52.01)	0.393	0.531
Female	88 (50.57)	454 (47.99)		
BMI (kg/m^2^)	25.78 (23.66, 27.77)	26.77 (24.11, 30.02)	-2.999	0.003
SBP (mmHg)	147.00 (137.00, 158.50)	142.00 (130.00, 154.75)	-3.415	<0.001
DBP (mmHg)	93.00 (84.00, 101.75)	89.00 (80.00, 99.00)	-3.560	<0.001
Serum Na (mmol/L)	143.00 (142.00, 145.00)	142.00 (141.00, 143.00)	-5.929	<0.001
Serum K (mmol/L)	3.30 (3.00, 3.60)	3.90 (3.60, 4.10)	-12.568	<0.001
Serum Na-to-K ratio	43.33 (38.98, 48.00)	36.67 (34.52, 39.44)	-12.625	<0.001
Serum Cl (mmol/L)	106.00 (104.00, 107.00)	105.00 (104.00, 107.00)	-1.029	0.304
Serum Ca (mmol/L)	2.26 (2.20, 2.31)	2.30 (2.23, 2.37)	-5.680	<0.001
AG	13.00 (11.62, 14.57)	14.10 (12.60, 15.60)	-4.859	<0.001
TC (mmol/L)	4.33 (3.70, 4.94)	4.73 (4.09, 5.49)	-5.230	<0.001
TG (mmol/L)	1.39 (0.99, 2.09)	1.68 (1.18, 2.32)	-3.566	<0.001
GLU (mmol/L)	4.80 (4.50, 5.40)	5.00 (4.50, 5.80)	-2.293	0.022
UREA (mmol/L)	4.40 (3.73, 5.40)	4.80 (4.10, 5.80)	-3.747	<0.001
CREA (μmol/L)	65.00 (55.00, 76.00)	64.00 (54.00, 75.00)	-0.777	0.437
UA (μmol/L)	319.00 (259.25, 374.75)	348.00 (287.25, 427.75)	-4.155	<0.001
Urine Na (mmol/L)	71.45 (49.15, 102.30)	82.45 (54.73, 113.68)	-2.915	0.004
24h Urine Na (mmol/24h)	160.13 (106.07, 232.25)	149.97 (102.67, 212.86)	-1.590	0.112
Urine K (mmol/L)	23.59 (17.28, 32.31)	19.55 (14.67, 27.57)	-4.717	<0.001
24h Urine K (mmol/24h)	55.51 (39.52, 73.88)	38.19 (28.69, 48.30)	-9.658	<0.001
Urine pH	6.50 (6.00, 7.00)	6.00 (5.50, 6.50)	-8.635	<0.001
Diabetes [n (%)]				
Yes	39 (22.41)	212 (22.41)	<0.001	0.999
No	135 (77.59)	734 (77.59)		
Hypokalemia [n (%)]				
Yes	60 (34.48)	115 (12.16)	55.568	<0.001
No	114 (65.52)	831 (87.84)		

**Figure 1:**
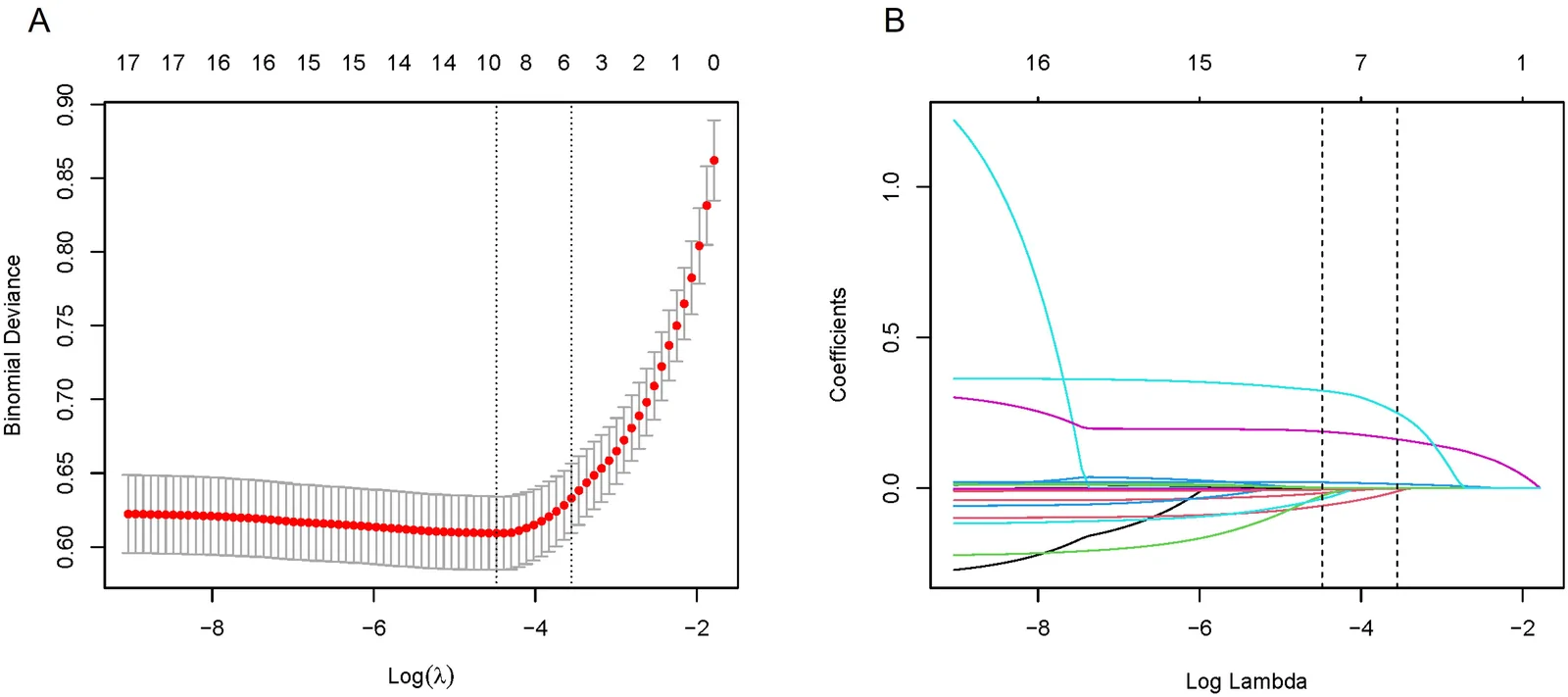
Lasso regression for the training set. **(A)** LASSO regression cross-validation. **(B)** LASSO regression path diagram.

Univariate logistic regression and stepwise backward multivariate logistic regression were performed on these six indicators. Ultimately, the same six indicators—serum sodium-to-potassium ratio, anion gap, uric acid, urine sodium, 24-hour urine potassium, and urine pH—were selected for constructing the final PA prediction model (**Tables 3**, **4**).

**Table 3:** Univariate logistic regression for the training set.

Variables	Univariate logistic regression				
	β	S.E	*Z*	*P*	OR (95%CI)
Serum Na-to-K ratio	0.239	0.019	12.611	<0.001	1.271 (1.224 ~ 1.319)
AG	-0.160	0.033	-4.876	<0.001	0.852 (0.799 ~ 0.909)
UA (μmol/L)	-0.004	0.001	-4.309	<0.001	0.996 (0.994 ~ 0.998)
Urine Na (mmol/L)	-0.007	0.002	-3.144	0.002	0.993 (0.989 ~ 0.997)
24h Urine K (mmol/24h)	0.033	0.004	8.473	<0.001	1.033 (1.025 ~ 1.041)
Urine pH	0.998	0.116	8.599	<0.001	2.712 (2.160 ~ 3.405)

**Table 4:** Multivariate logistic regression for the training set.

Variables	Multivariate logistic regression				
	β	S.E	*Z*	*P*	OR (95%CI)
Serum Na-to-K ratio	0.218	0.021	10.363	<0.001	1.243 (1.193 ~ 1.295)
AG	-0.098	0.042	-2.361	0.018	0.906 (0.835 ~ 0.983)
UA (μmol/L)	-0.003	0.001	-2.454	0.014	0.997 (0.995 ~ 0.999)
Urine Na (mmol/L)	-0.009	0.003	-3.485	<0.001	0.991 (0.985 ~ 0.996)
24h Urine K (mmol/24h)	0.024	0.004	5.392	<0.001	1.024 (1.015 ~ 1.033)
Urine pH	0.406	0.142	2.866	0.004	1.501 (1.137 ~ 1.983)

### 3.3 Construction and evaluation of the PA nomogram prediction model

The prediction model was visualized as a nomogram (**Figure 2**). The predictive performance of the model was assessed using ROC curves (**Figures 3A, D**). The AUC for the training set was 0.860 (95% CI: 0.825–0.894), with a sensitivity of 0.736 and a specificity of 0.876. The validation set achieved an AUC of 0.850 (95% CI: 0.800–0.899), sensitivity of 0.795, and specificity of 0.787. For the validation set, the AUC values of the PFK score and ARR were 0.718 (95% CI: 0.656–0.781) and 0.965 (95% CI: 0.943–0.987), respectively, with corresponding sensitivities of 0.846 and 0.936, and specificities of 0.469 and 0.923 (**Figure 4**). Notably, our predictive model showed significantly better diagnostic performance than the PFK score, as determined by the DeLong test (*P* < 0.001) (**Table 5**). The PFK score (14) was defined as follows: 1 point for female, 1 point for serum potassium < 3.5 mmol/L, and 1 point for urine pH > 7.0, with a maximum total score of 3 points.

**Figure 2:**
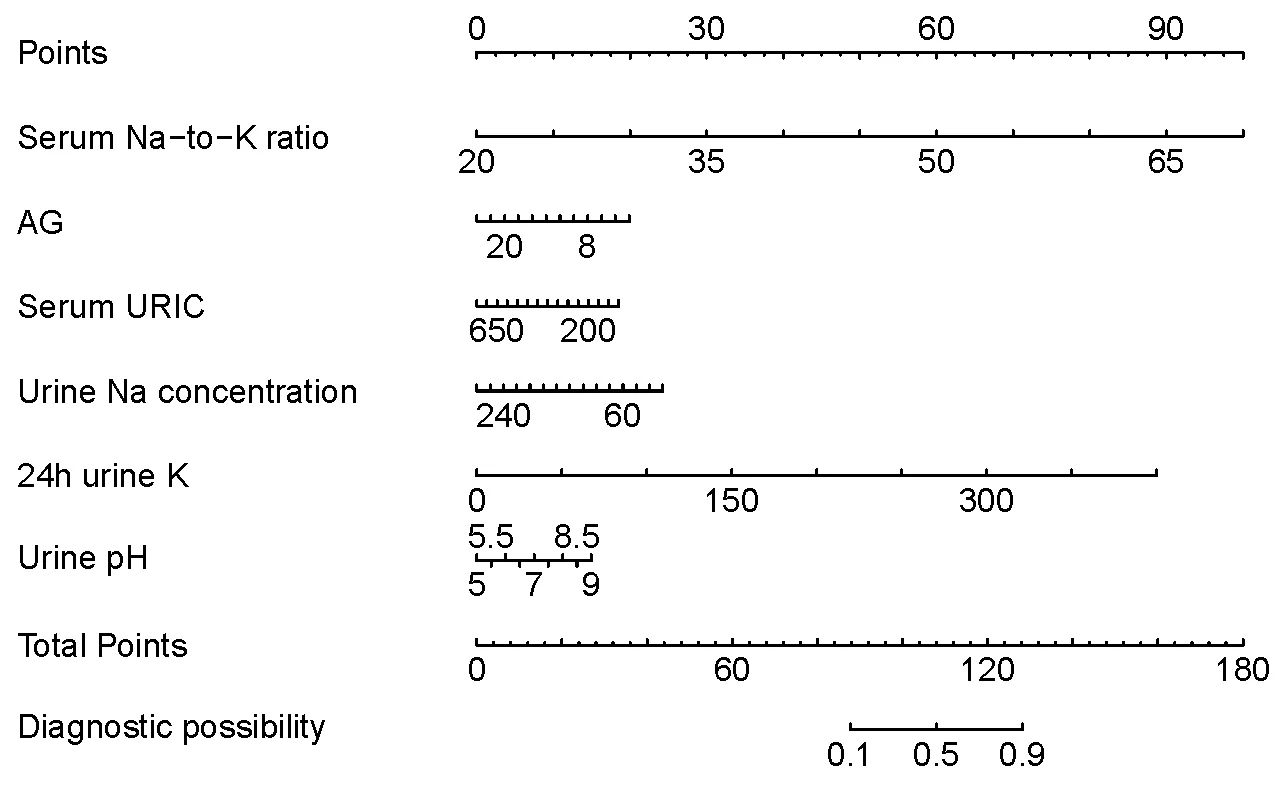
Nomogram of PA prediction model.

**Figure 3:**
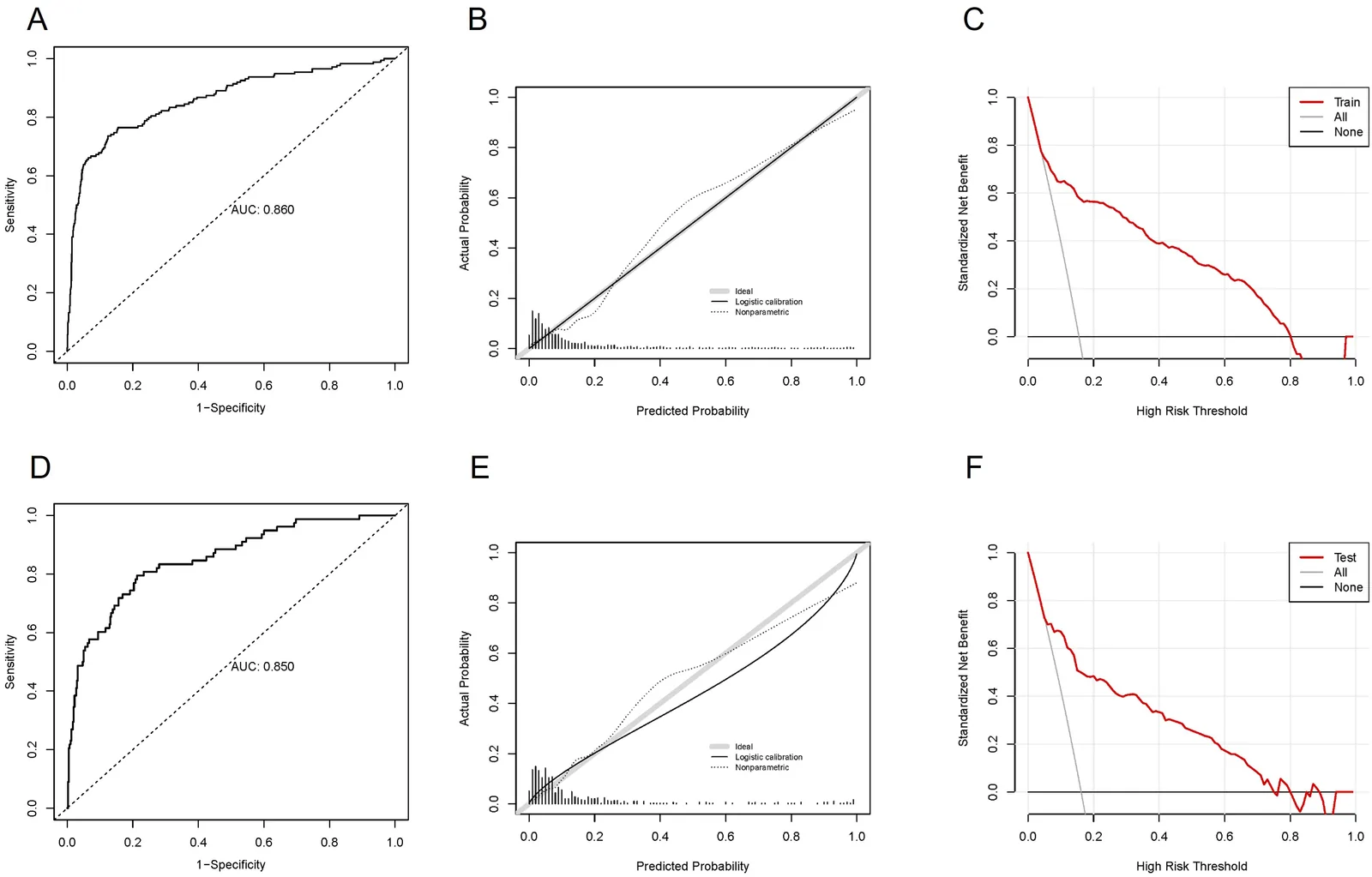
Validation of PA prediction model. **(A)** ROC curve for training set of prediction model. **(B)** Calibration curve for training set of prediction model. **(C)** Decision curve analysis (DCA) curve for training set of prediction model. **(D)** ROC curve for validation set of prediction model. **(E)** Calibration curve for validation set of prediction model. **(F)** DCA curve for validation set of prediction model.

**Figure 4:**
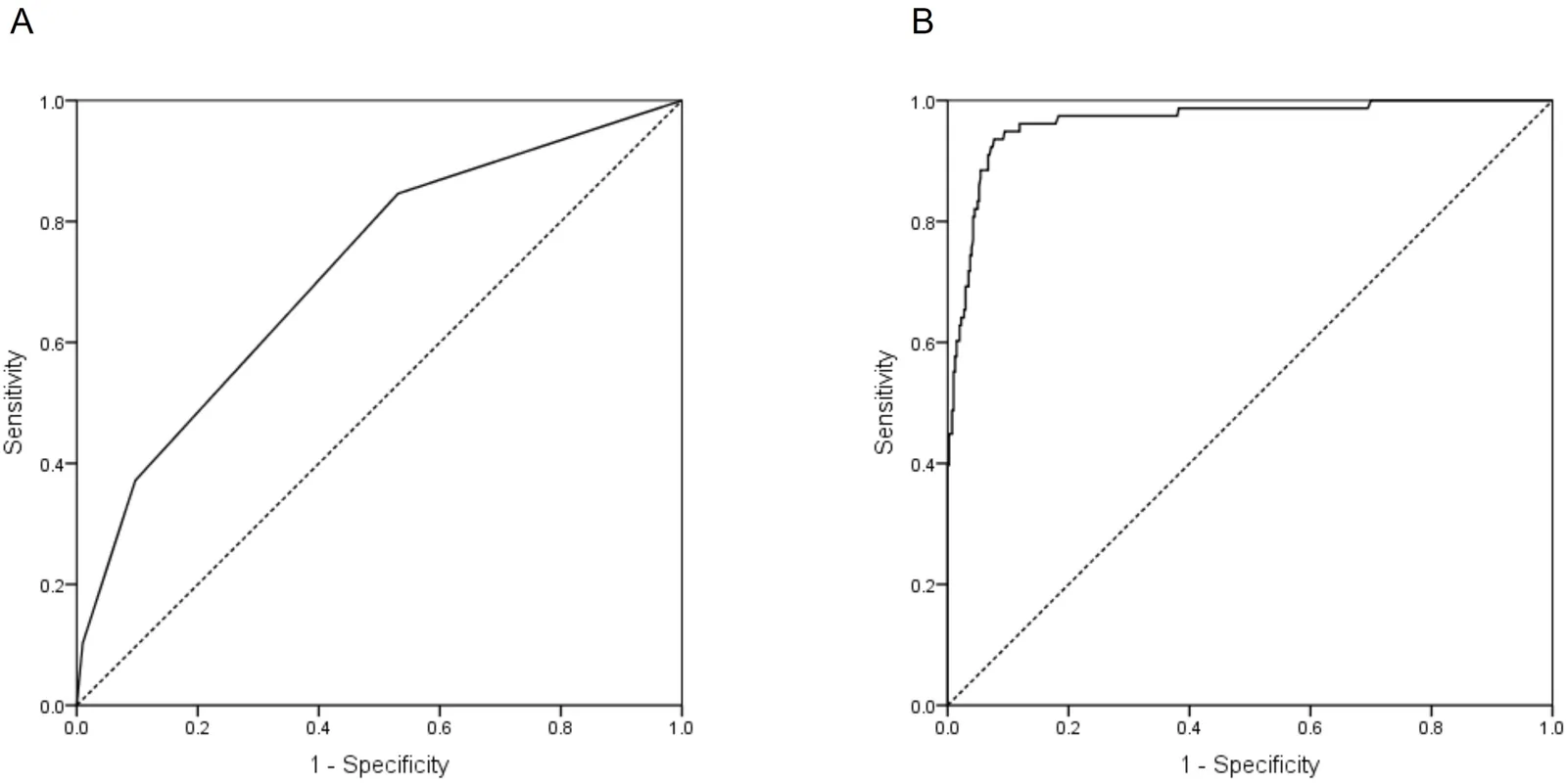
ROC curves for validation set PFK score and ARR. **(A)** ROC curve for validation set PFK score. **(B)** ROC curve for validation set ARR.

**Table 5:** DeLong test between nomogram, PFK score, and ARR.

Comparison	*Z*	*P*
Nomogram vs ARR	-4.974	<0.001
PFK score vs ARR	8.727	<0.001
Nomogram vs PFK score	4.457	<0.001

The Hosmer-Lemeshow test was used to assess the agreement between the model predictions and the actual results, yielding a *χ²* of 7.897 (*P* = 0.444) for the training set and 6.208 (*P* = 0.624) for the validation set, indicating good model fit. Calibration curves are shown in **Figures 3B, E**. Decision curve analysis (DCA) was employed to evaluate the clinical utility of the model in decision-making. DCA (**Figures 3C, F**) demonstrated that using this model to predict primary aldosteronism provided significant clinical benefit to patients. The predictive model showed good agreement with ARR in screening for PA (*Kappa* = 0.364, *P* < 0.001), which was better than the agreement between the PFK score and ARR (*Kappa* = 0.224, *P* < 0.001) (**Table 6**).

**Table 6:** Consistency between nomogram, PFK score, and ARR.

ARR	Nomogram		PFK score	
	+	-	+	-
+	71	33	90	14
–	92	285	190	187
*Kappa*	0.364		0.224	
*P*	<0.001		<0.001	

## 4 Discussion

PA was initially considered a rare cause of hypertension. However, with increasing research in recent years, its prevalence has risen annually. Despite this, PA screening remains suboptimal, largely due to the high technical demands of ARR measurement and poor patient compliance (6). To facilitate PA screening in hypertensive patients, we developed and internally validated a novel nomogram prediction model for PA based on six readily available indicators: serum sodium-to-potassium ratio, anion gap, uric acid, urine sodium, 24-hour urine potassium, and urine pH. This model may serve as a potential tool for risk stratification that may guide referral decisions in secondary or tertiary care settings, with future simplification needed for primary care adaptation. It is expected to improve patient compliance with PA screening and thereby promote the early diagnosis and treatment of PA.

As a mineralocorticoid, aldosterone plays a key role in maintaining extracellular volume homeostasis, blood pressure regulation, and serum potassium balance. In patients with PA, autonomous aldosterone secretion from the adrenal cortex leads to increased water and sodium retention, and enhanced potassium excretion, while suppressing the renin-angiotensin system. Clinically, this manifests primarily as hypertension and hypokalemia (15, 16). However, not all PA patients present with hypokalemia, despite excessive aldosterone secretion (17). Studies have reported a hypokalemia prevalence of approximately 37.5% in PA patients (3). In the present study, we observed hypokalemia in 34.48% of PA patients and 12.16% of EH patients (**Table 2**). Given the high prevalence of hypokalemia in PA, it is also of interest to examine the prevalence of PA among patients presenting with hypokalemia. Another study reported a PA prevalence of 28.1% among hypokalemic patients (18). To systematically investigate the relationship between serum potassium levels and PA, we comprehensively analyzed serum sodium, serum potassium, serum sodium-to-potassium ratio, and anion gap in both PA and EH patients. Additionally, alterations in serum electrolytes may influence urine electrolyte levels; therefore, we incorporated urine sodium and potassium into the predictive model. Our results indicated that elevated sodium-to-potassium ratio and 24-hour urine potassium, along with decreased anion gap and urine sodium, were independently associated with a higher predicted probability of PA. The molecular mechanisms underlying reduced serum potassium in PA patients remain incompletely understood. Studies indicate that somatic *KCNJ5* mutations occur in some aldosterone-producing adenomas, leading to structural and functional alterations in the *GIRK4* protein, which results in potassium loss and increased sodium permeability (19). Additionally, the *ATP1A1* gene may also participate in aldosterone regulation. Approximately 5.2% of patients with aldosterone-producing adenomas carry *ATP1A*1 mutations, which affect the potassium-binding site and reduce the affinity of the ion pump for potassium ions (20).

Studies have shown a close association between PA and metabolic syndrome (21, 22). Elevated aldosterone and reduced serum potassium increase the risk of diabetes (23, 24). Therefore, in the present study, we analyzed blood glucose, serum lipid levels, and diabetes status in PA and EH patients. The results showed no significant differences in these parameters between the two groups, which is consistent with previous reports (25). Furthermore, studies indicate that PA patients have lower uric acid levels than EH patients, and both groups show a decreasing trend in serum uric acid with rising plasma aldosterone levels and declining renin activity. PA patients often have concomitant renal impairment (26–28). Therefore, we included renal function parameters in our statistical analysis of PA and EH patients. Results indicate that serum uric acid levels were independently associated with PA. Specifically, lower uric acid levels were associated with higher odds of PA. This finding may be related to elevated aldosterone levels in PA patients. High aldosterone induces sodium retention and potassium excretion, thereby increasing blood volume and renal blood flow perfusion, which in turn enhances uric acid excretion and ultimately lowering serum uric acid. Furthermore, studies have reported varying degrees of proximal renal tubular damage in PA patients, which reduces uric acid reabsorption and consequently leads to lower serum uric acid levels (29, 30).

In summary, this study analyzed clinical laboratory parameters associated with PA and developed a novel prediction model incorporating serum sodium-to-potassium ratio, anion gap, uric acid, urine sodium, 24-hour urine potassium, and urine pH, presented as a nomogram. Internal validation of the predictive model demonstrated good discriminatory ability based on the ROC curve. The calibration curves and DCA indicated sound predictive performance and clinical benefit, and the *Kappa* test showed satisfactory agreement with ARR. For clinical institutions lacking aldosterone and renin testing capabilities, all parameters included in this model are routine serum and urine tests available in clinical laboratories. Therefore, this model can effectively assists clinicians in screening for PA, enabling efficient diagnostic evaluation for patients with limited access to medical resources, and thereby promoting early diagnosis and treatment of PA.

Several limitations of this study should be acknowledged. First, the study population was derived from hypertensive inpatients at a single tertiary university hospital, and the cohort already included patients with indications for PA screening (e.g., resistant hypertension, hypokalemia, adrenal incidentaloma, family history of PA, and obstructive sleep apnea). This high-risk profile reflects a referral-biased population and may not be representative of the broader hypertensive population encountered in primary care settings. Consequently, the model’s performance in unselected hypertensive patients requires further validation. Further multicenter studies with external validation in community-based or primary care cohorts are needed before the model can be generalized to lower-risk populations. Second, some patients in the study had comorbidities such as pneumonia, cholecystitis, urinary tract infections, or kidney stones, and the medications they received for these conditions may have interfered with the study results. Third, although the nomogram demonstrated good predictive performance in our cohort, its current implementation relies on 24-hour urine collections and multiple electrolyte measurements, which may limit its direct applicability in resource-constrained primary care settings. Future studies should explore whether simplified versions of the model—using more readily available variables—could achieve comparable performance, thereby enhancing its utility in primary healthcare settings.

## Data Availability

The original contributions presented in the study are included in the article/supplementary material. Further inquiries can be directed to the corresponding author.
